# Timely, consistent, transparent assessment of market access evidence: implementing tools based on the HTA Core Model® in a pharmaceutical company

**DOI:** 10.1017/S0266462318003653

**Published:** 2019

**Authors:** Pierre Ducournau, Cornelia Irl, Iain Tatt, Mary McCarvil, Marlene Gyldmark

**Affiliations:** 1 MORSE – Health Technology Assessment Group, F. Hoffmann-La Roche; 2 Department of Biostatistics, Genentech Inc.

**Keywords:** Technology assessment, Biomedical, Economics, Pharmaceutical, biomedical technology, Healthcare sector, Reference standards

## Abstract

**Objectives:**

Evidence requirements and assessment methods access differ between health technology assessment (HTA) agencies. The HTA Core Model^®^ provides a standardized approach to HTA, targeting evidence sharing and collaboration between participating HTA bodies. It is fit for purpose from an industry perspective and was used by pharmaceutical company Roche to develop a framework for internal assessment of evidence required for market access and coverage/reimbursement (“access evidence”).

**Methods:**

Tools were developed to systematically scope, assess, plan, and summarize access evidence generation. The tools were based mainly on the first four HTA Core Model^®^ domains and rolled-out in selected development teams in 2017. Five months after full implementation, the impact of tools was assessed in an internal survey.

**Results:**

Systematic access evidence generation started with the Access Evidence Questionnaire, to scope evidence requirements and identify evidence gaps. Findings were summarized in the Access Evidence Metric, which assessed the alignment of available/planned evidence against HTA bodies’ requirements and developed scope mitigation strategies. The Access Evidence Plan was then used to plan and document (additional) evidence generation. Once generated, evidence was summarized in the Access Evidence Dossier. A survey of twenty-seven Roche employees involved in evidence generation showed that the tools made discussions around access strategies and evidence more efficient and transparent.

**Conclusions:**

The HTA Core Model^®^ provided a useful framework around which to optimize internal evidence generation and assessment. The benefits of using a standardized HTA approach in industry mirror those expected from implementing the HTA Core Model® in HTA agencies.

Making novel healthcare technologies available is a complex process that requires careful assessment of the technology from the perspective of various stakeholders in healthcare, for example, patients, providers, manufacturers, and payers (1–3). In a first step, marketing authorization is granted, based on the quality, safety, and efficacy of the technology (4). In a second step, coverage and reimbursement decisions are made, which also account for the value of a novel technology (1–3). The evidence documenting the value of a technology from a health technology assessment (HTA) or payer perspective includes but goes beyond the “regulatory evidence” (which documents quality, safety, and efficacy), and is referred to as “access evidence” in this study. Access evidence establishes a technology's value to patients, healthcare providers, and healthcare payers for example by demonstrating the benefit of the technology over standard of care and current clinical practice. Examples may include improved long-term clinical outcomes, better quality of life in patients, or reduced treatment costs in health economic analyses (1–3;5).

Access evidence is usually generated by manufacturers to be assessed and appraised in HTA, which in turn informs coverage and reimbursement decisions (1;6). There is, however, substantial variation between jurisdictions and settings in how HTA is conducted, what evidence is required and considered, and how value decisions are made (5–8). As a result of this variation, duplicated work may reduce the efficiency of HTA processes and increase their costs while delaying patient access to healthcare technologies (5–7;9–11). From the perspective of manufacturers, between-setting variation in HTA leads to a loss not only of efficiency in generating and preparing relevant access evidence but also of predictability of HTA outcomes (9;10).

To provide a framework for increased transferability of HTA and stronger collaboration on HTA across settings, the HTA Core Model^®^ (“the model”) was developed by the European Network for Health Technology Assessment [EUnetHTA] (12). The model provides an ontology, which is structured around nine domains relevant to HTA (Health Problem and Current Use of Technology, Description and Technical Characteristics of Technology, Safety and Clinical Effectiveness, Costs and Economic Evaluation, Ethical Analysis, Organisational Aspects, Social Aspects, Legal Aspects) (13). While full HTA would use all nine domains, a framework was developed for Rapid Relative Economic Assessments (REAs) that included only the first four domains. For manufacturers, the model also provided an opportunity to support standardized, internal generation and assessment of the access evidence required in reimbursement submissions for new products. As different HTA agencies put different emphasis on different aspects of value and access evidence, early and consistent planning of access evidence generation is necessary to avoid insufficient or late evidence, as well as duplication of work and inefficient resource allocation.

In 2013, pharmaceutical company F. Hoffmann-La Roche (Roche) set up a joint project with EUnetHTA to evaluate if and how the model could support standardized assessment of market access evidence in industry (“market access” denotes making a healthcare technology available to patients and achieving reimbursement from healthcare payers for the technology). The HTA Core Model^®^ was chosen as it is a well-established framework that has undergone extensive use by HTA agencies, particularly in Europe, and as its structure was anticipated to include most aspects relevant to access evidence generation. The model was expected to be of value by providing a useful assessment framework and common vocabulary that could contribute to discuss efficiently the profile and value of a technology both within the company and with external stakeholders (14). Roche aimed to implement a standardized process for optimizing the internal assessment of access evidence and developed four access evidence tools (AEx tools), based on the HTA Core Model^®^, to guide consistent and timely consideration of access evidence throughout the development lifecycle of a healthcare technology (used to denote both drugs and medical devices).

## Methods

The development of a standardized evidence assessment process was informed by the internal evaluation of the HTA Core Model^®^ (14) and a review of HTA processes. Model domains and their evaluation were compared with HTA and reimbursement guidelines as well as previous experiences with HTA and reimbursement submissions by staff working in market access and reimbursement, to identify the following key aims of evidence generation: (i) Scoping disease- and indication-specific evidence requirements for payers and HTA agencies, (ii) Assessing the existing evidence generation plan and identification of potential evidence gaps that may pose a risk for market access, (iii) Developing and evaluating different options to address evidence gaps and planning the generation of additional evidence, (iv) Summarizing the access evidence for ready use in HTA submissions.

For each aim, an AEx tool was developed as a structured template with clear guidance to product development teams on how to proceed systematically and transparently at each stage of access evidence generation. The AEx tools were based on the first four domains of the HTA Core Model^®^, namely the Health Problem and Current Use of Technology, Description and Technical Characteristics of Technology, Safety and Clinical Effectiveness, which are used for Rapid REAs (15). These four domains were considered important to characterize and discuss the profile and value of a technology in different country settings (14). Where appropriate, specific elements from the Costs and Economic Evaluation and Ethical Analysis domains were also included in the AEx tool design. The other domains were too setting-specific for the global AEx documents. Completion of these three domains, if required and relevant, remained with affiliates using setting-specific guidance.

An internal survey of stakeholders from different functions and teams was conducted in October and November 2017, approximately 5 months after full implementation of the AEx tools and processes were made available to teams in all disease areas across the company. The aim of the survey was to provide a detailed assessment of strengths and weaknesses of the tools. Interviewees were staff working at managerial levels and as team leaders in a range of disease areas, departments, and groups, such as pharmaceutical development, medical affairs, pricing and market access, and country affiliates. Interviewees were sampled from attendance lists of AEx tools development workshops and an internal register of teams where AEx tools were already in use. Potential interviewees were contacted by the first author by means of email, which explained the purpose of the survey, and an interview was arranged. Interviews, which lasted 30–60 minutes each, were conducted with open questions to elicit opinions from interviewees on the overall access evidence process, AEx tools, and areas of improvement. Survey results were summarized quantitatively, with descriptive statistics, and qualitatively, with key narratives distilled from responses to assess if AEx tools helped to achieve the aims of access evidence generation.

## Results

### Design and Contents of AEx Tools

#### Scoping the Access Evidence Landscape: The Access Evidence Questionnaire

The Access Evidence Questionnaire (AEQ) was developed to scope access evidence requirements and identify evidence gaps. Based on HTA Core Model^®^ domains 1–4, the AEQ was structured in five sections and designed to guide the development team through an extensive assessment of evidence requirements for the disease and technology of interest (Table 1). For all responses, documentation of information sources was required to guarantee transparency of the AEQ for internal planning and discussions. Two product development stage-specific versions of the AEQ were developed, namely a short version to be completed before the start of early phase of development and a full version to be completed before the start of pivotal clinical trials. Relative to the full version, the short version of the AEQ contained a reduced set of those items that were deemed relevant during early development. Examples of items removed from the short version included the impact of the technology on non–disease-specific mortality or anticipated changes in resource use, which were addressed, however, in the full version before pivotal trials were initiated.

**Table 1.** Structure and Contents of the Access Evidence Questionnaire

Section	Aim	Contents	Example questions
General project questions	Summarize market access strategy	• Project information • Stage of development • Launch strategy • Clinical development plan	• What phase are the pivotal clinical trials to support regulatory filing? • What are the proposed primary endpoints for the trial?
Population	Identify the target population and unmet needs	• Unmet needs addressed by the new therapy • Clinical characteristics of the disease • Differences in trial and target populations • Subgroups relevant for payers or HTA agencies	• What are the unmet needs that will be addressed for this indication and target population? • Is the target population well defined in clinical guidelines/literature, and are there market differences (e.g. in size of the target population)? • What is the prognosis for this disease? • How will the baseline risk of the patient population be estimated from the clinical trial?
Standard of care and comparator	Understand the current and future treatment landscape	• Current treatment options • Future treatment options • Overview of comparator(s)	• By market, what are the currently approved treatments in the target indication? • What new treatment options are expected to be available at the time of launch? • What comparator is being selected in the proposed Phase 2 or Phase 3 trial and what is the rationale for this selection? • By market, will the proposed comparator be appropriate from a payer/HTA body perspective? Why?
Efficacy and safety	Understand clinical effectiveness and safety	• Mortality • Morbidity • Patient-relevant endpoints and HRQoL • Effectiveness estimates and measures • Safety	• Will disease-specific mortality be a key endpoint of the planned studies? • Will utility data be collected as part of the clinical development program? If so, will a generic instrument, e.g. EQ-5D, be used for collecting utilities? • Will efficacy be estimated after end of treatment? If so, how will this be done? • If there are safety/tolerability issues, what is the incidence, severity and duration of the harm
Additional domains for access evidence consideration	Provide overview of access evidence for additional value drivers	• Study characteristics • Dosing and route of administration • Resource requirements • Other domains of interest (as appropriate)	• What is the dosing and route of administration of the standard of care and the other most commonly used treatment options? • Are the resource requirements anticipated to differ by market? • If appropriate: ethical, organizational, cost and economic, patient environment and social or legal aspects to be considered in line with domains specified by the HTA Core Model®

HRQoL, health-related quality of life; HTA, health technology assessment.

#### Assessing the Planned Evidence Generation: The Access Evidence Metric

The Access Evidence Metric (AEM) was developed to summarize findings from the AEQ and assess the alignment between the available/planned evidence and the evidence required to achieve the desired benefit rating by a payer or HTA agency (Table 2). For each setting, the desired benefit rating was defined as the rating associated with the target reimbursement rate on the jurisdiction- or setting-specific scale, for example, “major” or “considerable additional benefit” in Germany or “major” or “important clinical added value” in France (16;17). For each drug and indication, the AEM was updated regularly to reflect both internal evidence generation activities and external changes, for example, recent decisions by payers or HTA bodies on new treatments. The AEM was used to identify potential market access risks and inform subsequent access evidence planning.

**Table 2.** Process for completing the Access Evidence Metric

Step	Aim
1	Set the desired overall benefit rating
2	Identify the evidence requirements associated with the desired benefit rating
3	Identify the available/planned access evidence for each domain
4	Assess the alignment of the required with the available/planned evidence for each domain
5	Assess the influence of each domain on the overall benefit rating
6	Assess the overall alignment of the required with the available/planned evidence
7	Develop strategies to deal with the evidence misalignments (if any)
8	Update the Access Evidence Metric with outcomes from access evidence planning

For each domain considered in the AEQ (e.g., efficacy or quality of life), the available/planned evidence was classified to be in “full alignment,” “partial alignment,” or “lack of alignment” with the required evidence. Where misalignments were identified, the discrepancy between requirements and available/planned evidence is described. In addition, the importance of each domain for assessing benefit and value and for achieving the desired benefit rating was classified as “essential,” “important,” or “unimportant.” The available/planned evidence was considered fully aligned overall with evidence requirements only if full alignment was achieved in all essential and important domains. Following this overall assessment, potential market access risks could be identified, enabling the development of strategies to overcome misalignments and assess their likely impact. Finally, the AEM was updated with outcomes from access evidence planning (see the third AEx tool).

#### Planning (Additional) Access Evidence Generation: The Access Evidence Plan

The Access Evidence Plan (AEP) was developed to plan and document access evidence generation. Completion of the tool took the form of a workshop, during which all relevant functions and teams provided structured input to the planning of (additional) evidence generation. In the AEP, access evidence needs and gaps, as identified in the AEM, were discussed to develop mitigation strategies as well as their risks and trade-offs. Upon finalization of the AEP, the AEM was updated accordingly.

#### Summarizing Access Evidence: The Access Evidence Dossier

The Access Evidence Dossier (AED) was developed to provide a global evidence summary by integrating evidence from all sources. Accompanied by comprehensive internal guidance, the AED served the basis for global value guidance and HTA submissions. The structure of the AED closely followed that of the HTA Core Model^®^ for Rapid REA and focused on the first four domains (15). Upon completion, the AED was made available to affiliates to support setting-specific submissions aimed at demonstrating value to payers and HTA bodies and achieving desired benefit ratings.

### Completion, Lifecycle, and Remit of AEx Tools

**Figure 1.**
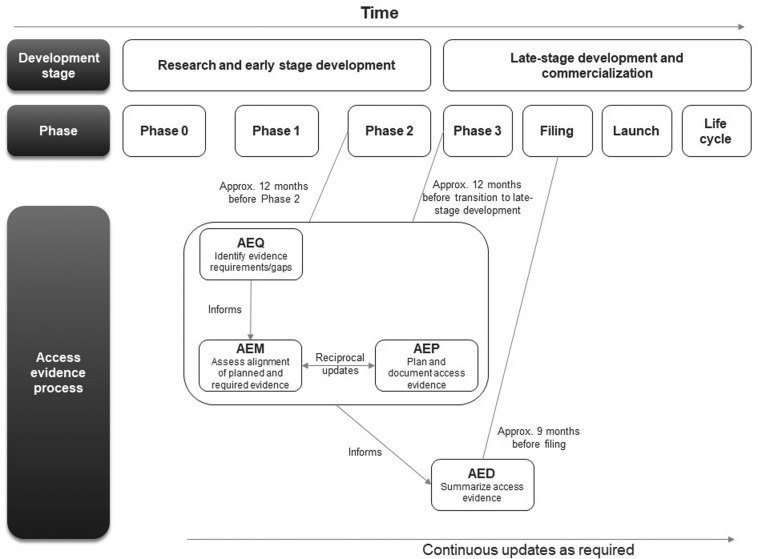
Workflow for completing and updating the Access Evidence Questionnaire, Metric, and Plan. AED, Access Evidence Dossier; AEM, Access Evidence Metric; AEP, Access Evidence Plan; AEQ, Access Evidence Questionnaire.

The first AEx tool to be completed was the AEQ, which informed the AEM (Figure 1). The AEM was then used to inform the AEP, while outcomes of access evidence planning were in turn used to update the AEM. The AEM was submitted to internal governance and review committees for weighing of market access risks and endorsement. Review of the AEM could also lead to changes clinical development plans which would then be reflected in updates to the AEM and AEP. The AED was completed at a later stage, once all evidence was available (which might occur several years after the initial development of the accompanying AEx tools and endorsement of the AEM).

The lifecycle of AEx tools generally began once an indication has been identified for the drug under development. Tools were updated regularly and reflected important development milestones such as the design of pivotal studies or major changes in strategy or external circumstances. Initiation of work on the AED was scheduled approximately 9 months before regulatory submission for marketing authorization at the European Union (EU) level. The AED was updated after initial completion to accommodate new or changed data as well as feedback and requests from payers and HTA bodies.

The remit of AEx tools comprised all drugs developed by Roche during research and early development as well as during late stage confirmatory development and commercialization and across all development phases, from before phase 1 clinical trials to product launch and management of its lifecycle (e.g., regarding real-world uptake and use) in each market. Notably, AEx tools were designed to complement and support, not to replace, existing processes for strategic planning, evidence generation and submission preparation. AEx tools were developed for evidence assessments in the United States and key Roche markets in the EU (France, Germany, Italy, United Kingdom) but can also accommodate assessments for other countries.

### Roll-out and Internal Assessment of AEx Tools

The tools and guidance on their use were rolled out across several product development teams throughout the company. In addition, procedures establishing responsibilities for and timing of AEx tools completion and updates over the product lifecycle were implemented. Simultaneously, an online platform was developed on which to store the AEx tools for ready access by affiliates and development teams.

Implementation of the AEx tools and the standardized evidence assessment process at Roche began in January 2017 in selected product teams. Teams volunteered for participation and were selected by the lead investigator to reflect a range of different disease areas. By July 2017, twelve teams across the portfolio used the tools and followed the newly established process. By December 2017, the tools and process were implemented for thirty drugs/indications, all in the countries for which AEx tools had been developed. The tools were integrated into templates and processes for decision governance bodies. In addition, AEx tools were made available on the online platform to all involved global and affiliate teams and stakeholders.

In a survey of users and cross-functional stakeholders, the impact of AEx tools was assessed and areas for improvement were identified. A total of twenty-two interviews were conducted with twenty-seven Roche employees involved in development and use of the AEx tools. Most interviews were individual interviews, while four interviews were conducted as group interviews with an entire team. In group interviews, responses were collected separately for each interviewee as interviewees did not necessarily agree on all items. Of the interviewees, most (43 percent) came from pharmaceutical development functions, followed by participants from commercial functions (27 percent) and affiliates (20 percent), while the remainder were from research and early development (10 percent).

Overall, the AEx tools and processes were considered to provide the expected value, that is, more transparency and clarity for decision makers and affiliates around access evidence planning and generation, alignments on market access strategies, and more efficient discussions around evidence generation. All interviewees reported that the overall process for generating evidence was working well and achieved the aims of access evidence generation, especially as it fostered alignment across teams with regard to evidence requirements, gaps, planning, and documentation (Table 3). Two-thirds of interviewees stated that the access evidence process had a direct effect on drafting clinical development plans and target product profiles while 19 percent declared that the process had an impact but would have to be balanced against local requirements. The AEx tools were considered by 95 percent of interviewees to positively affect evidence generation, particularly with regard to structuring discussions around evidence planning as the current state of and additional requirements for evidence generation were transparent at any time to all involved staff.

**Table 3.** Results of the Roche stakeholder Survey on Access Evidence Tools

Statement	Percent of interviewees (*N* = 27)	Comment, additional information
*Areas that work well*		
Access evidence process provides value to the molecule lifecycle	100	Value of process due to, among other reasons, the cross-functional alignment on access evidence
Access evidence tools have a positive impact on access evidence generation	95	Positive impact due to, among others, the structure of evidence generation provided by the tools
*Impact of the access evidence process on clinical development plans and target product profiles*		
Access evidence process has an impact	66	The Access Evidence Plan and associated discussions around trade-off with regard to access evidence generation were particularly emphasized
Access evidence process has an impact but needs to be balanced against what is required and feasible locally	19	—
Access evidence process does not have an impact	5	It was suggested that only minimal requirements for regulatory approval could be considered in the process
Uncertain if the access evidence process has an impact	10	Interviewees providing this response were from teams too early in the access evidence process to identify a clear impact
*Areas for improvement*		
More communication needed around the access evidence process	30	More communication on the purpose and aims of the access evidence process, also in relation to other submission-relevant documentation, was suggested to be helpful to increase acceptance by all stakeholders
Improvements to the Access Evidence Dossier	70	The length and overlap of the Access Evidence Dossier with other filing documents were suggested as areas to improve the dossier

An area of improvement was the length of the AED (approximately equal to a Rapid REA), in particular given its partial overlap with other submission documents. It was suggested to integrate into the AED the possibility to link to other documents, to further reduce duplication of work. Seventy percent of interviewees thought that the communication on the purpose of access evidence generation and the tools worked well. Thirty percent suggested that more communication would be helpful to increase internal understanding and commitment to the access evidence process.

## Discussion

The present study reports on the development and implementation of a standardized approach to assess evidence generation in a large pharmaceutical company. Several tools for scoping, assessing, planning, and summarizing evidence were developed and built into the product development lifecycle to provide a transparent and clear structure for evidence generation across teams and functions. Development of the AEx tools was informed by an internal assessment of the HTA Core Model^®^. For implementation in AEx tools, HTA Core Model^®^ domains were adapted to specific Roche requirements to increase their applicability and usability. Compared with the model, domains in the AEx tools were shorter and more targeted, with elements considered redundant or less relevant from an industry perspective left out or combined, respectively. A similar approach to adapting the HTA Core Model^®^ by reducing domains and re-shuffling assessment elements was chosen by HTA agencies, for example, in the development of an HTA framework in Lombardy (18). These adaptations were performed to tailor the HTA Core Model^®^ to specific institutional requirements, mirroring the experience at Roche (19;20).

The initial roll-out of the evidence assessment process and AEx tools covered teams in multiple therapeutic areas. Both the process overall and the tools were considered by users and process leaders to work well and add value. Discussions around access evidence, for example, were judged to have become more structured and efficient both within and across teams. Challenges in using the tools were an initial lack of familiarity and partial overlap of the tools with existing process and submission requirements. In addition, more communication around the intention and role of the AEx tools was considered helpful. This feedback was used to inform further development of the tools and the planned company-wide roll-out to ensure efficient implementation and acceptance by internal stakeholders (21;22).

Developed in response to the absence of a standardized HTA framework in key markets, the AEx tools and the process overall are anticipated to have several benefits for Roche, some of which have already been realized. First, the timely consideration of evidence requirements increases the efficiency of evidence generation within the company. A standardized approach to scoping, assessing, planning, and summarizing evidence, with information and evidence shared in real-time across involved teams, is associated with a lower risk of missing evidence or duplicating work. With coordination costs reduced due to standardized tools and central documentation of evidence and with time saved due to duplicated work avoided, resources can be freed up and used, for example, on accelerating evidence generation or extending the communication with external stakeholders. In addition, a standardized approach used across different disease areas is likely to improve quality control capacities and staff mobility: as there is a common, company-wide access evidence vocabulary, experience with access evidence generation in one area becomes applicable in another.

Second, from the perspective of internal stakeholders and decision makers, the transparency of a standardized process to assess access evidence is useful to inform internal product development and investment decisions. Evidence gaps that might affect market access, in addition to costs and development time associated with additional evidence generation, can be identified and documented early on in the drug development lifecycle. Consequently, the transparency of decision making is expected to increase in line with a shared and better understanding of the strategies and status of the available/planned evidence. In addition, evidence needs can be documented systematically while internal expectations and evidence assessments can be compared with assessments and decisions by payers and HTA bodies. Such a comparison is anticipated to provide feedback loops and, thereby, opportunities to improve internal evidence assessment and generation further.

Third, the standardized approach, based on the HTA Core Model® and centered on the AEx tools, can support the move toward a digitized, more automated production of HTA evidence within the company. A structured framework can be developed into an online platform to enable collaboration as well as information exchange and storage across teams and functions. From the perspective of Roche, the foundation for collaborative, Web-based evidence generation has been laid by the development and roll-out of the *#TAg* platform, on which AEx tools and evidence are stored. Benefits of this digitized approach are anticipated to include faster evidence generation and sharing with reduced transaction costs as well as more efficient collaboration (23).

In conclusion, a systematic approach and a set of tools designed for scoping, assessing, planning, and summarizing access evidence for submission to payers and HTA agencies were implemented at Roche. The tools were developed based on an assessment of the HTA Core Model^®^, which was adapted to the specific requirements of Roche. The tools are currently implemented in several disease areas and contribute to structuring assessments of and discussions around evidence generation. Over the coming years, the tools will be refined further and implemented throughout the company.

These results show that it is possible to implement a standardized process for access evidence assessment, based on the HTA Core Model^®^ framework, throughout product development inside a pharmaceutical company. Similar to the benefits that can be derived from standardization of HTA requirements across settings, a standardized approach to assessing evidence and its generation within a company is likely to be associated with efficiency gains and improved decision making and contributes to providing relevant evidence for demonstrating value.
